# Quality check: concordance between two monitoring systems for postoperative organ/space-surgical site infections in rectal cancer surgery. Linkage of data from the Catalan Cancer Plan and the VINCat infection surveillance programme

**DOI:** 10.1186/s12957-024-03410-9

**Published:** 2024-05-25

**Authors:** Carlota Matallana, Miguel Pera, Eloy Espin-Basany, Sebastiano Biondo, Josep M Badia, Enric Limon, Miquel Pujol, Borja de Lacy, Luisa Aliste, Josep M Borràs, Paula Manchon-Walsh

**Affiliations:** 1 Catalonian Cancer Strategy, Health Department, Hospital Duran i Reynals Hospital, Av. Gran Via de l’Hospitalet, 199-203- 1ª planta,08908 L’Hospitalet de Llobregat, Barcelona, Spain; 2 Universitat Autònoma de Barcelona. Plaça Cívica, Bellaterra, Barcelona, 08193 Spain; 3 Department of General and Digestive Surgery, Hospital del Mar, Passeig Marítim 25-29, Barcelona, 08003 Spain; 4 Department of General and Digestive Surgery Department, Institute of Digestive and Metabolic Diseases (ICMDM), Biomedical Research Centre (CIBERehd), Hospital Clinic, IDIBAPS, University of Barcelona, Barcelona, Spain; 5 Colorectal Surgery Unit, Vall d’Hebrón University Hospital, Pº de la Vall d’Hebron, 119-129, Barcelona, 08035 Spain; 6 Department of General and Digestive Surgery-Colorectal Unit, Bellvitge University Hospital, C/Feixa Llarga, s/n, 08907 L’Hospitalet de Llobregat, Barcelona, Spain; 7 Biomedical Research Institute of Bellvitge (IDIBELL), Universitat de Barcelona, C/Feixa Llarga, s/n, 08907 L’Hospitalet de Llobregat, Barcelona, Spain; 8 Department of Surgery, Hospital General de Granollers, Av Francesc Ribas 1, Barcelona, 08402 Granollers Spain; 9 School of Medicine, Universitat Internacional de Catalunya, Barcelona, Spain; 10 Departament de Salut, VINCat Programme - Surveillance of Healthcare Related Infections in Catalonia, Barcelona, Spain; 11 Department of Public Health, Mental Health and Mother–Infant Nursing, Faculty of Nursing, University of Barcelona, Barcelona, Spain; 12 Centro de Investigación Biomédica en Red de Enfermedades Infecciosas CIBERINFEC, Instituto Carlos III, Madrid, Spain

**Keywords:** Surgical Site infection, Surgical Wound Infection/prevention & control*, Cohort studies, Rectal Surgery* / adverse effects, Rectal cancer, Surgical Wound infection / prevention & control, Databases concordance

## Abstract

**Background:**

The Catalan Cancer Plan (CCP) undertakes periodic audits of cancer treatment outcomes, including organ/space surgical site infections (O/S-SSI) rates, while the Catalan Healthcare-associated Infections Surveillance Programme (VINCat) carries out standardized prospective surveillance of surgical site infections (SSI) in colorectal surgery. This cohort study aimed to assess the concordance between these two monitoring systems for O/S-SSI following primary rectal cancer surgery.

**Methods:**

The study compared O/S-SSI incidence data from CCP clinical audits versus the VINCat Programme in patients undergoing surgery for primary rectal cancer, in 2011-12 and 2015-16, in publicly funded centres in Spain. The main outcome variable was the incidence of O/S-SSI in the first 30 days after surgery. Concordance between the two registers was analysed using Cohen’s kappa. Discordant cases were reviewed by an expert, and the main reasons for discrepancies evaluated.

**Results:**

Pooling data from both databases generated a sample of 2867 patients. Of these, O/S-SSI was detected in 414 patients—235 were common to both registry systems, with satisfactory concordance (κ = 0.69, 95% confidence interval 0.65–0.73). The rate of discordance from the CCP (positive cases in VINCat and negative in CCP) was 2.7%, and from VINCat (positive in CCP and negative in VINCat) was 3.6%. External review confirmed O/S-SSI in 66.2% of the cases in the CCP registry and 52.9% in VINCat.

**Conclusions:**

This type of synergy shows the potential of pooling data from two different information sources with a satisfactory level of agreement as a means to improving O/S-SSI detection. ClinicalTrials.gov Identifier: NCT06104579. Registered 30 November 2023.

## Introduction

Colorectal cancer (CRC) is the third most common malignant neoplasm and the fourth cause of cancer death worldwide [1]. In the absence of tumour dissemination, surgical excision is currently the main therapeutic option with curative intent. Surgical site infections (SSI) are among the most common postoperative complications, associated with increased morbidity, mortality, and healthcare-related costs [2, 3]. These infections can occur following any surgical procedure; however, CRC surgery has the highest incidence after elective abdominal procedure (9–20%)^4,5^ .

In CRC surgery, organ/space surgical site infections (O/S-SSI), which are mainly secondary to an anastomotic leak (AL), are one of the most serious complications [6]. The reported incidence of AL after colorectal surgery is 3–21% and is higher in distal rectal anastomoses and in patients undergoing emergency operations [7]. About half of O/S-SSI are considered preventable, and epidemiological surveillance with feedback providers is considered an important component of strategies implemented to reduce their rates [8, 9].

Several studies have shown that anastomotic leak and subsequent organ space - surgical site infection (O/S-SSI) are also associated with higher rates of tumor recurrence and cancer-specific mortality [10, 11]. The severity of the postoperative infection has also been correlated with the increased risk of recurrence [12]. However, this effect has not been found in other studies [13–15]. The question of whether AL contributes to disease recurrence remains controversial and requires further investigation [16].

The healthcare system in Catalonia (Spain) monitors and reports SSI in cancer patients through two principal mechanisms. First of all, the Catalan Cancer Plan (CCP) is a Health Department structure that aims to improve quality of care for cancer patients by means of periodic auditing of outcomes with real-world data and feedback to professionals [17]. Data mainly come from the review of medical documentation by a team of external auditors, with full coverage of patients receiving treatment in the public system; one indicator included in these compulsory audits is the occurrence of O/S-SSI^18^. On the other hand, the Catalan Healthcare-associated Infections Surveillance Programme (VINCat) is a network spanning all across centres, which has been conducting surveillance in colorectal surgery since 2008^5^. The two systems thus operate with an important difference: the CCP audits are mandatory and cover all cancer surgeries performed in centres funded through the public healthcare system, while VINCat is a voluntary registry programme among participating centres [19]. The selected cases and methods used to detect O/S-SSI may therefore vary substantially between auditing systems.

This retrospective population-based cohort study aims to assess the concordance between clinical audits of the CCP and the VINCat registry as a preliminary step to further studies on CRC recurrence and O/S-SSI.

## Methods

### Study design, setting and patients 

This population-based cohort study took place in Catalonia, Spain (7.7 million inhabitants), where the public agency CatSalut funds 68 centres in the Catalan health system (SISCAT) that provide hospital care and participate to some extent in cancer care. The study compares data from the two databases in the two time periods in which CCP audits were conducted: 2011–2012 and 2015–2016.

### Study outcomes, variables, definitions

The primary outcome was the occurrence of O/S-SSI. In the CCP database, the categorisation of a case as O/S-SSI was assessed on the basis of descriptions and diagnostics in discharge reports and electronic medical record notes.

In the VINCat database, identification of O/S-SSI cases was done by the infection control teams (ICTs) of each hospital according to the definitions of the Centers for Disease Control and Prevention (CDC) and the National Healthcare Safety Network (NHSN) classification of operative procedures [20]. On this basis, an O/S-SSI in rectal surgery is defined as an infection occurring within 30 days of the surgical procedure and involving any part of the body deeper than the fascial/muscle layers that is opened or manipulated during surgery. In addition, the patient must present at least one of the following associated events: a purulent drainage from a drain placed into the organ/space; (an) organism(s) identified from fluid or tissue in the organ/space by a culture or non-culture-based microbiological testing method, performed for the purpose of clinical diagnosis or treatment; an abscess or other evidence of infection involving the organ/space, detected on gross anatomical exam or histopathologic exam; or definitive or equivocal evidence of infection on imaging test.

### Data source

 The CCP carries out periodic mandatory audits of SISCAT hospitals through the review of the electronic health records by a team of external auditors. The audits are conducted every 3–4 years, a time interval that is considered sufficient to detect potential changes in the quality of care provided to rectal cancer patients. CCP have chosen to include as the most recent data those corresponding to the 15–16 audit in order to achieve a follow-up of more than 5 years from the time of first surgery with curative intent. Data were drawn from the records of patients with primary rectal cancer who had undergone curative surgery in SISCAT hospitals. One of the indicators included in these audits is the occurrence of O/S-SSI, which was assessed on the basis of descriptions in discharge reports and electronic medical records.

Currently, almost all centres integrated in SISCAT participate in the VINCat surveillance system (55/68 hospitals). This programme conducts active surveillance of SSIs in elective colorectal surgery at public and private hospitals. Data are collected by the local multidisciplinary ICTs and transmitted electronically to the coordination centre. Mandatory active surveillance after discharge is conducted until postoperative day 30 using a multimodal approach, including electronic review of medical records (with access to out-of-hospital care notes), verification of readmissions, verification of emergency department visits, and review of microbiological and radiological data. On a regular basis, audits of the data provided by the hospitals are carried out to ensure the accuracy of the programme data [5]. All cases of rectal surgery included in the VINCat system during the two periods analysed were included in the study. Data extraction was approved by the CCP Board.

### Linkage of databases

 As a preliminary step to the present study, VINCat conducted an internal validation of its data in 2011 by means of an interobserver concordance study. A total of 220 cases from a representative sample of centres were validated by a trained team from the VINCat study group in colorectal surgery. The decision of the validating team prevailed over that of the hospital. Inter-observer concordance was established using the Kappa index and was classified as very good in most centres.

Table 1 shows the criteria for the CCP audits, the standardized methodology of VINCat selection programme and the selection criteria for the present study.

To correlate the results of the two databases, patients diagnosed with O/S-SSI were selected from each database, and the two lists were compared for the concordance analysis. Accordingly, the incidence of O/S-SSI was assessed during the first 30 days after surgery, and any O/S-SSI that was not identified by both systems was reviewed by a colorectal surgeon. Patients were classified according to whether their records matched (concordant) or did not match (discordant) between the two databases.

**Table 1:** Case selection criteria for systems monitoring organ/space-surgical site infections in Catalonia and for the present study

Inclusion criteria	Exclusion criteria
Catalan Cancer Plan	
• Elective rectal cancer surgery performed in 2011–2012 and 2015–2016 in publicly financed healthcare centres (CatSalut)	• Procedures performed in centres with private financing and foundations • Surgeries for benign colorectal diseases • Palliative surgeries • < 18 years old
**VINCat programme**	
• Elective resection of the colon or rectum • Wound class 2 (Clean-contaminated) and 3 (Contaminated) cases • Minimum of 100 consecutive procedures per year per hospital or continuous monitoring throughout the year for centres that perform fewer than 100 procedures per year	• Emergency surgery • Peritonitis at the time of intervention (wound class 4). • Patients who underwent multiple procedures during the same surgery • Centres that performed < 10 surgical procedures annually
**Present study**	
• Patients > 18 years old • Eligible patients with tumour ≤ 13 cm from anal verge, as measured by MRI • Primary adenocarcinoma • Oncological resection with curative intent (R0-R1) • Cancer stages: I-II-III • Surgery techniques: TaTME, RAR, APR, Hartmann, total proctocolectomy, pelvic exenteration	• Transanal local resection (TEM/TEO/TAMIS) • Emergency rectal surgeries • Presence of metastases found in the diagnostic process or during the surgical procedure • Recurrence of the disease treated before the study period • Non-resectable tumour or palliative surgery • Patients operated on in private centres

*APR* abdominoperineal resection, *MRI* magnetic resonance imaging, *RAR* rectal anterior resection, *TAMIS* transanal minimally invasive surgery, *TaTME* transanal total mesorectal excision, *TEM* tranasnal endoscopic microsurgery, *TEO* transanal endoscopic operation

### Statistical analysis

 Infection rates were expressed as cumulative incidence, that is, the crude percentage of operations resulting in SSI/number of surgery procedures. A person-level linkage was performed between the two databases. Concordance between the two registers was analysed using Cohen’s Kappa, with a 95% confidence level. The results are analysed using the IBM SPSS Statistics package (V21.0).

### Ethical issues

The confidentiality of patients’ personal data was strictly guaranteed in accordance with European regulations through the registry of patient identifiers in a database independent from that of the clinical data used in the study and held by the CCP. This study was approved by the research ethics committee of Bellvitge University Hospital (PR286/21).

This research has been registered with ClinicalTrials.gov Identifier: NCT06104579 (https://register.clinicaltrials.gov/prs/app/action/SelectProtocol?sid=S000DSJ9&selectaction=Edit&uid=U0004FX4&ts=4&cx=8joidi. The study has been reported in accordance with the RECORD statement [21], an extension of the STROBE statement [22].

## Results

**Fig. 1:**
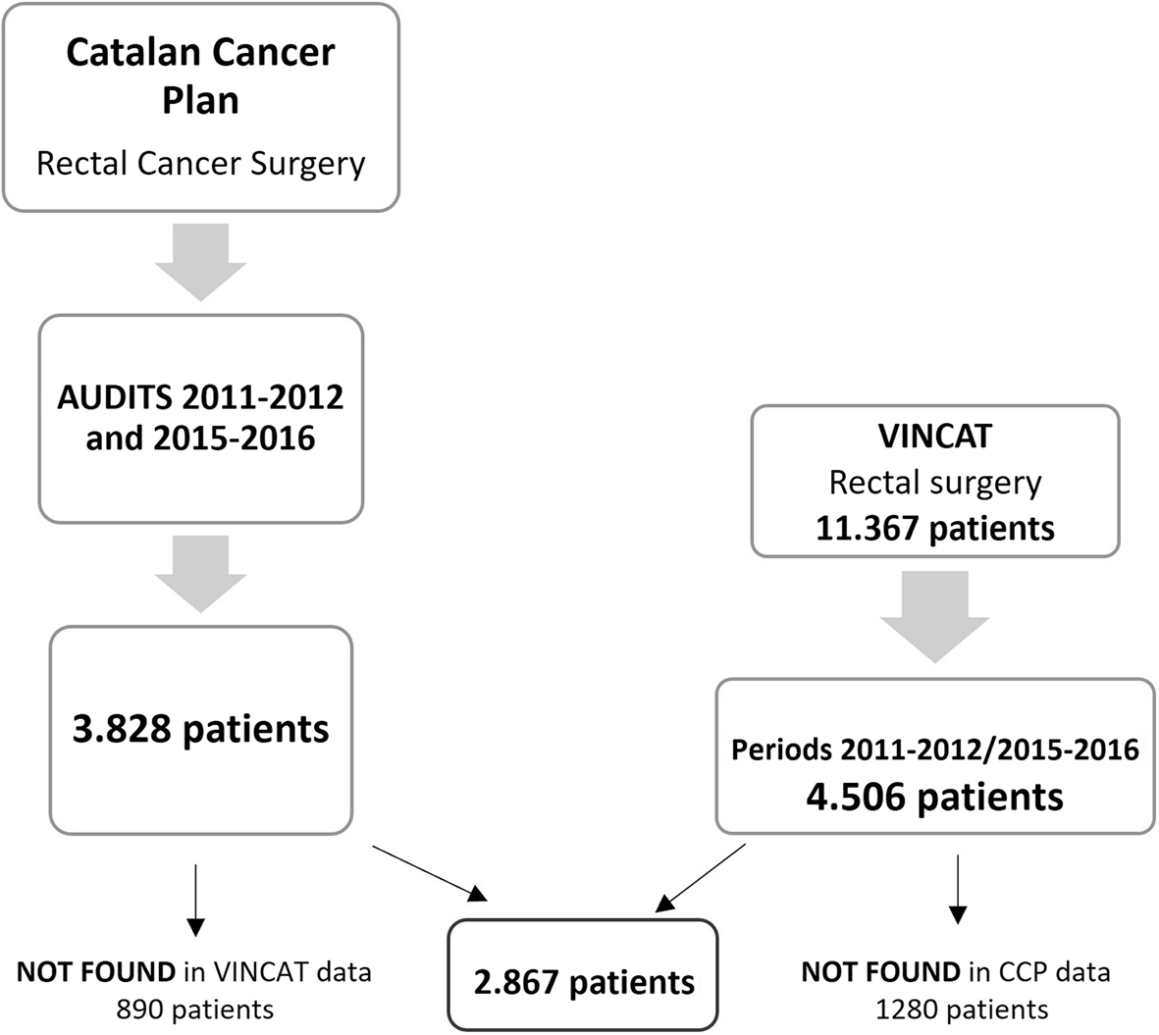
Study flowchart.  VINCAT: Catalan Healthcare-associated Infections Surveillance Programme

The search identified patients from the two registries (Fig. 1). After applying exclusion criteria, 4,506 patients were included from the VINCat database and 3,828 from CCP audits during the 2011–2012 and 2015–2016 periods. Cross-referencing both databases yielded 2,867 common cases (study population), while 890 cases were found only in the CCP registry and 1,280 cases only in VINCat.

The baseline characteristics of each of these groups are described in Table 2, and Table 3 shows the descriptive baseline characteristics of the overall cases in the study population.

Concordance between the included registry systems is shown in Table 4.

**Table 2:** Overall baseline clinical characteristics in patients included in the VINCat and Catalan Cancer Plan registry systems

Variable		*N*	%
Age, years, mean (SD)		68.28 (11.23)	
Sex	Male	1911	66.7
	Female	956	33.3
ASA	ASA I	151	5.3
	ASA II	1551	54.1
	ASA III	990	34.5
	ASA IV	73	2.5
	ASA V	0	0.0
	Unknown	102	3.6
Type of surgery	Urgent	10	0.3
	Elective	2857	99.7
Surgical approach	Laparotomy	970	33.8
	Laparoscopy	1834	64.0
	Unknown	63	2.2
	Total	2867	

*ASA* American society of anesthesiologists classification, *CCP* Catalan cancer plan, *VINCat* Catalan Healthcare-associated Infections surveillance programme, *SD* standard deviation

**Table 3:** Baseline clinical characteristics in patients to both Catalan Cancer Plan and VINCat databases

		VINCat			CCP	
		*N*	%		*N*	%
Sex	Male	789	61.6		580	65.2
	Female	491	38.4		310	34.8
ASA	ASA I	75	5.9		36	4.0
	ASA II	743	58.0		426	47.9
	ASA III	434	33.9		330	37.1
	ASA IV	27	2.1		39	4.4
	ASA V	0	0.0		0	0.0
	Unknown	1	0.1		59	6.6
Type of surgery	Emergency	12	0.9		82	9.2
	Elective	1268	99.1		808	90.8
Surgical approach	Laparatomy	598	46.7		409	46.0
	Laparoscopy	670	52.3		359	40.3
	Unknown	12	0.9		122	13.7
	Total	1280			890	

*ASA* American society of anesthesiologists classification, *VINCAT* Catalan healthcare-associated infections surveillance programme, *CCP* Catalan cancer plan

**Table 4:** Concordance between CCP and VINCat population data related to organ/space-surgical site infections (O/S-SSI), *n* (%)

		CCP		TOTAL
		No O/S-SSI	O/S-SSI	
**VINCat**	**No O/S-SSI**	2453 (85.6)	102 (3.6)	2555 (89.1)
	**O/S-SSI**	77 (2.7)	235 (8.2)	312 (10.9)
**TOTAL**		2530 (88.3)	337 (11.7)	2867 (100)

*CCP* Catalan cancer plan, *VINCat* Catalan healthcare-associated infections surveillance programme, *O/S-SSI* organ/space surgical site infections

**Fig. 2:**
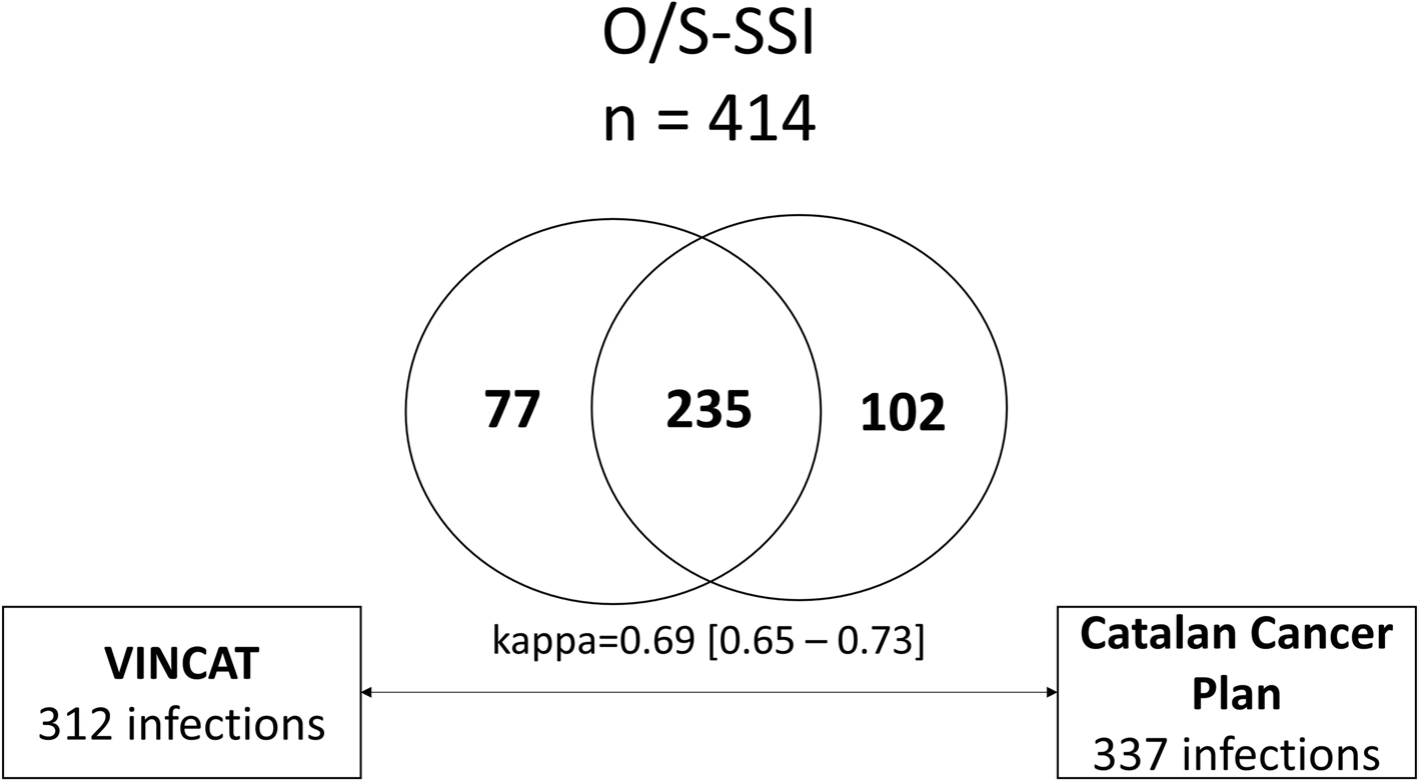
Venn diagram to illustrate linkage process and concordance between CCP and VINCat population data related to organ/space-surgical site infection. VINCAT: Catalan Healthcare-associated Infections Surveillance Programme. CCP: Catalan Cancer Plan.

Of the 414 total patients in whom an O/S-SSI was detected, 235 were identified by both databases (Fig. 2). Regarding discordances, out of the 2867 patients studied, the CCP did not detect 77 patients (2.7%) registered as having O/S-SSI in VINCat, while VINCat missed 102 (3.6%) cases detected by the CCP (Fig. 2).

Discordant cases were reviewed by an external colorectal surgeon and an epidemiologist from the CCP; Table 5 presents the results of this analysis. Twenty-six out of 312 cases (8.3%) in the VINCat registry and 48 out of 337 (14.2%) in the CCP registry were misclassified as O/S-SSI. Thus, detection of real O/S-SSI was better in the VINCat registry system. The concordance between the two systems was satisfactory (κ = 0.69, 95% CI 0.65–0.73).

**Table 5:** Results of external review of discordant cases

Final determination	O/S-SSI detected by VINCat but not CCP	O/S-SSI detected by CCP but not VINCat
**Definitive O/S-SSI,*n*(%)**	**51 (66.2)**	**54 (52.9)**
Hospital readmission	17	11
Confirmation of O/S-SSI	34	43
**No O/S-SSI, n (%)**	**26 (33.8)**	**48 (47.1)**
Other infection^a^	5	19
O/S-SSI presented in > 30 days	3	2
Others^b^	0	7
Confirmation of no O/S-SSI	18	21
**Total**	**77 (100)**	**102 (100)**

*O/S-SSI* organ/space surgical site infections, *VINCat* Catalan healthcare-associated infections surveillance programme, *CCP* Catalan cancer plan

^a^13 cases due to a deep incisional infection in APR procedure

^b^Haematoma, bleeding, stoma dysfunction, etc

## Discussion

In this population-based cohort study, we observed satisfactory concordance between two detection systems for O/S-SSI in rectal cancer surgery. Both have a wide population coverage and a long history of clinical monitoring [17]. The CCP undertakes periodic audits to improve the quality of cancer care and standardize clinical practice. However, cases treated in private centres, which account for around 13% of colorectal cancer surgeries, are not included. On the other hand, VINCat is a voluntary surveillance programme that collects data from 59 public and private hospitals but does not analyse all rectal surgery cases, as each hospital can include only the first 100 cases each year. Prospective surveillance is performed by a trained infection control team at each hospital to ensure appropriate data collection, in line with a detailed operational definition document which was generated and shared with all network hospitals [5].

After applying appropriate selection criteria and combining the databases, we found that 6.2% of cases were discordant. First, there were 102 patients (3.6% of the overall study cohort) identified by CCP as O/S-SSI but not by VINCAT. An external review confirmed that only 54 presented a real O/S-SSI. A closer analysis revealed that one reason that CCP failed to detect these cases is the confusion between the classification of an organ/space and a deep-incisional wound infection, mainly in the abdominoperineal resection procedure. This discordance may also be explained by the heterogeneous diagnostic criteria of O/S-SSI followed by the hospitals included in CCP and the self-reporting by each centre participating in the VINCat system. Self-reported data are at risk of several potential biases, despite validation activities. On the other hand, the CCP seemingly failed to detect 77 patients with O/S-SSI (2.7%), 51 of whom were later confirmed as having true O/S-SSI on external review. This problem could be due to the retrospective nature of the data collection and the variability in the quality of the O/S-SSI information in the clinical records. Furthermore, the CCP data reflects variability in the use of standardized protocols to detect and diagnose postoperative infections. In fact, the long history of the system means that data collection spans different changes in the protocols implemented in the Healthcare system.

Readmission to the hospital, and in turn the existence of a second clinical record, was a common reason for failing to detect O/S-SSI in both register systems. At the same time, some unexplained discordances also remain. Thus, our recommendation is to focus future research on identifying O/S-SSI, standardizing criteria, and implementing a common detection protocol across all centres.

In this study, the incidence rate of detected O/S-SSI was almost 11%. Of note, the rates of postoperative infections reported in the literature reflect the presence of SSI in general (including superficial and deep incisional surgical site infections), rather than organ/space infections specifically. In this line, Ali-Mucheru et al. estimated an incidence rate of 24% in their study from 2020^3^. In 2023, Malheiro et al. reported a cumulative incidence of SSI (including O/S-SSI) of 16.8% out of 11,129 procedures, 61% of which is attributed to all risk factors and 31% to modifiable variables [23]. Accordingly, efforts to monitor and reduce SSI are a major quality improvement priority for patients, payers, and providers [24]. The present study shows how two different information sources can be combined, with a favourable concordance, contributing to efforts to reduce SSI rates in colorectal elective surgery [25].

The study has some limitations, beginning with the retrospective nature of the clinical data and the length of time elapsed. From 2011 to 2016 (the periods selected for the analysis) changes in clinical practices and data collection methods may have occurred, which could affect the detection of O/S-SSI and the concordance between registry systems. Secondly, unlike the audits carried out by the CCP, which are mandatory to guarantee high-quality cancer care, the participation in the VINCat surveillance programme is voluntary. Finally, the fact that the discordant cases were evaluated by an external surgeon and an epidemiologist and not cross-checked with other specialties could be considered a self-assessment bias in the results.

Two main strengths can also be highlighted. As far as we know, this is the first study to combine two large registry systems in order to enhance the monitoring process of a postoperative infective complication. Secondly, all the discordant cases were externally analysed and evaluated to identify the probable causes of errors and find practical solutions. Every measure contributing to the improvement of such surveillance programmes is necessary if the Spanish healthcare system expects to improve SSI outcomes and, in turn, the quality of patient care [26, 27].

In conclusion, the level of concordance between the two systems for O/S-SSI detection is satisfactory, validating the quality of information. This type of synergy shows the potential of combining data from two different information sources with the common objective of improving the quality of cancer surgery [28]. In our opinion, the combination of external audits with high-quality information and their application for controlling and monitoring postoperative infective complications should be a priority. Obtaining a cohort of patients with a confirmed diagnosis of O/S-SSI by cross-referencing both databases will allow future studies to correlate the development of O/S-SSI and local recurrence in this type of surgery.

## Data Availability

The data that support the findings of this study are available from Catalan Cancer Plan and VINCat but restrictions apply to the availability of these data, which were used under license for the current study, and so are not publicly available. Data are however available from the authors upon reasonable request and with permission of the Technical Committees of Catalan Cancer Plan and VINCat (Catalan Department of Health).
